# Neurofibroma of the Palate

**DOI:** 10.1155/2014/898505

**Published:** 2014-04-17

**Authors:** Tirumalasetty Sreenivasa Bharath, Yelamolu Rama Krishna, Govind Rajkumar Nalabolu, Swetha Pasupuleti, Suneela Surapaneni, Suresh Babu Ganta

**Affiliations:** ^1^Department of Oral Pathology, Vishnu Dental College, Bhimavaram, Andhra Pradesh 534 202, India; ^2^Department of Oral Surgery, Vishnu Dental College, Bhimavaram, Andhra Pradesh 534 202, India

## Abstract

Neurofibroma is a benign peripheral nerve sheath tumor comprising variable mixture of Schwann cells, perineurial-like cells, and fibroblasts. Neurofibroma may occur as solitary lesion or as part of a generalised syndrome of neurofibromatosis or very rarely as multiple neurofibromas without any associated syndrome. There are two distinct variants of neurofibromatosis type I and type II. We present a case of neurofibroma of the hard palate associated with neurofibromatosis type I. The diagnosis of the lesion was made based on the clinical findings, family history, histopathology, and immunohistochemistry. Literature was reviewed and different types of neurofibroma, their incidence and frequency in the oral cavity, its association with neurofibromatosis, clinical manifestations, histopathologic characteristics, immunohistochemical analysis, behaviour, treatment, and recurrence are discussed.

## 1. Introduction

Neurofibroma is a benign peripheral nerve sheath tumor, composed of a variable mixture of Schwann cells, perineurial-like cells, and fibroblasts as well as cells with intermediate features between these cells [1]. Neurofibromas of the oral cavity are presented as a submucosal, nontender, discrete mass. The tongue, buccal mucosa, and vestibular area are common sites and posterior mandible is the most common intraosseous location [1]. Neurofibroma may occur as solitary lesion or as part of a generalised syndrome of neurofibromatosis (von Recklinghausen's disease) or very rarely as multiple neurofibromas without any association with neurofibromatosis syndrome. They can be further divided based on their clinical presentation into localized or solitary growth, diffuse discrete or multiple nodules, and plexiform types [2].

The frequency of isolated neurofibromas unassociated with neurofibromatosis in the oral cavity is uncertain [3]. 4–7% of patients affected by neurofibromatosis display oral manifestations [4]. We report a case of neurofibroma of the hard palate associated with the syndromic NF-1 seen in a female patient and reviewed the literature related to various aspects of this condition.

## 2. Case Report

A 38-year-old female referred to the Department of Oral and Maxillofacial Surgery, Vishnu Dental College, with a chief complaint of swelling in the right side of palate since 2 years and associated pain since 1 month. The patient first noticed the swelling two years ago, which was of insidious onset and since then it had grown slowly to attain the present size. After an asymptomatic phase of 2 years the patient had started to experience mild, intermittent, dull aching pain in the swelling 1 month ago. The past medical and dental histories were not contributory. Family history revealed that her father also had same kind of palatal swelling and multiple nodules all over the body and now her 17-year-old daughter started developing multiple nodules all over the body.

**Figure 1:**
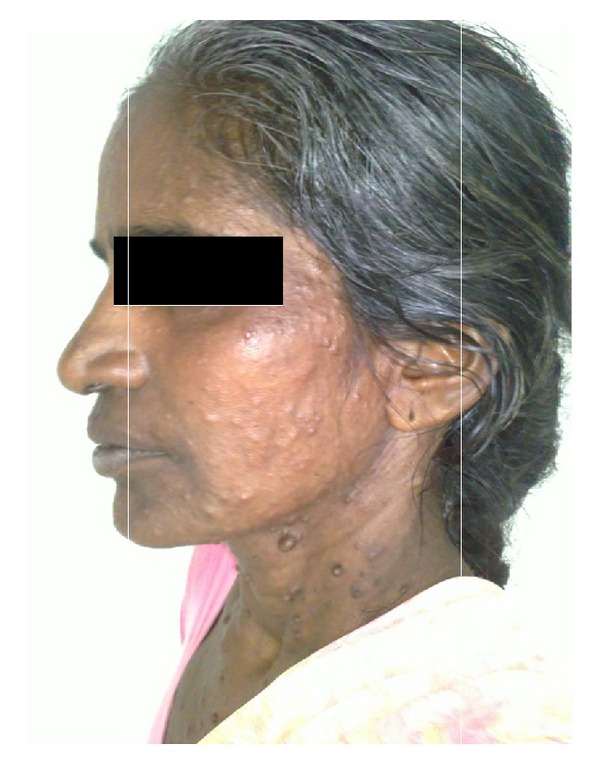
Diffuse multiple brownish black pigmented nodular masses of various sizes spread all over the body.

General examination revealed diffuse multiple brownish black pigmented nodular masses of various sizes spreading all over the body (Figure 1). On palpation, these noninflammatory nodules varied in consistency, from soft to firm. Ophthalmological examination revealed the presence of Lisch nodules and early onset of cataract, with no signs of glaucoma.

Lymph node examination revealed palpable right submandibular lymph node which is mobile, approximately 1 × 1 cm, nontender, and firm in consistency.

**Figure 2:**
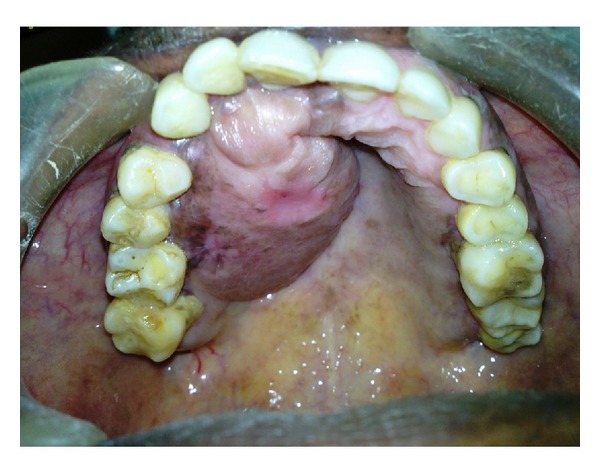
Solitary, oval, well-defined palatal swelling.

Intraoral examination revealed a solitary, oval, well-defined pedunculated swelling in the upper right palate extending from 11, 12 attached gingiva to 16 distal region, 1 cm away from the midpalatal suture. It measures approximately 2 × 3 cm in size and is irregular in shape and mucosa over the swelling appears pink in colour (Figure 2). On palpation there was no local rise in temperature or tender, fluctuant, and variable consistency. Differential diagnosis of the intraoral swelling includes lipoma, fibroma, schwannoma, and hemangioma.

The patient was subjected to radiographic investigation. The panoramic and posterior-anterior skull view did not reveal any evidence of bone involvement.

Based on clinical presentation of the generalised diffuse discrete multiple pigmented skin nodules, Lisch nodules in the eyes and intraoral well-circumscribed tumour growth, and family history of first-degree relatives, a provisional diagnosis of neurofibroma with neurofibromatosis type I was made.

After an informed written consent, an excisional biopsy was performed under local anesthesia for histopathological and immunohistochemical conformation of the clinical diagnosis.

## 3. Histopathology

The excised specimen macroscopically appeared as an oval, well-defined soft tissue mass measuring 2 × 3 cm, yellowish pink in colour, and its consistency varied from soft to firm area. The cut surface appeared glistening, greyish white in appearance, and its consistency varied with the presence of soft areas interspersed with firm nodular regions.

**Figure 3:**
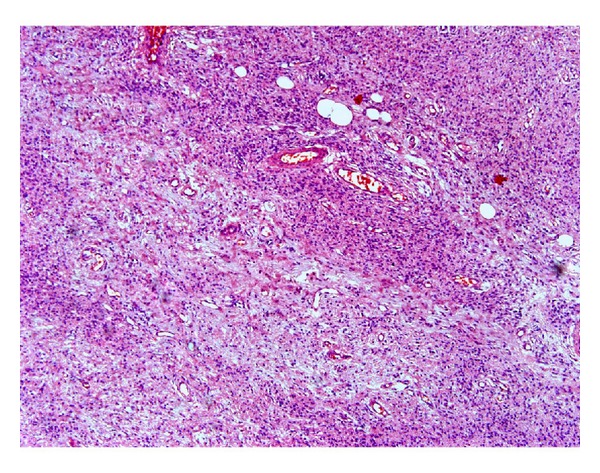
Fibrocellular connective tissue predominantly elongated spindle shaped with wavy and bent nuclei.

**Figure 4:**
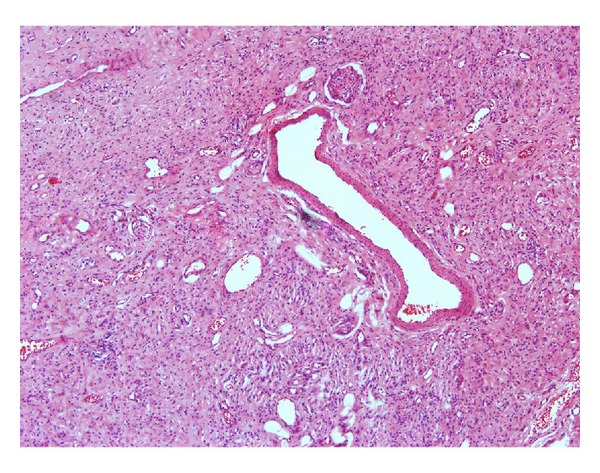
Peripheral nerve tissue within the perineural sheath and dilated blood capillaries.

Hematoxylin and eosin stained soft tissue section showed a benign neoplasm of the fibrous connective tissue exhibiting partial encapsulation. The connective tissue is highly cellular, interspersed with areas of nerve bundles. The cells are elongated spindle shaped with wavy and bent nuclei (Figure 3). They are separated by fine and wavy collagen fibers. Myxomatous areas are also seen. Peripheral nerve tissues within the perineural sheath are evident within the fibrous connective tissue (Figure 4). Few mast cells, dilated blood vessels, entrapped adipocytes, and mucous acini are evident all over the lesional area.

**Figure 5:**
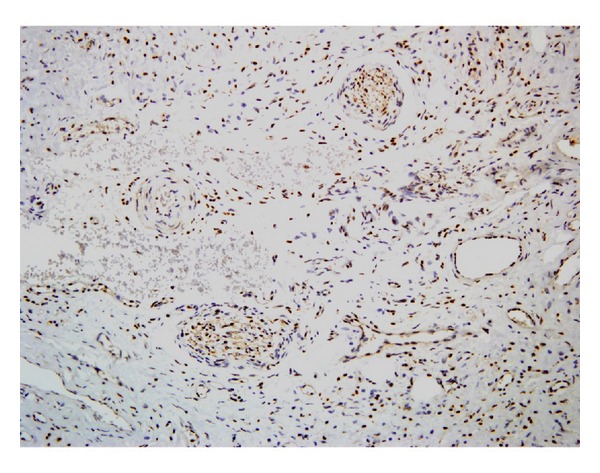
S-100 immunopositivity.

The lesional tissue section showed positive immunoreactivity to S-100 (Figure 5), neuron-specific enolase, and vimentin and negative immunoreactivity to desmin and pancytokeratin. Based on both histopathological and immunohistochemical analysis the final diagnosis was consistent with the clinical diagnosis of neurofibroma of the palate associated with neurofibromatosis type I. Patient was kept under observation for periodical review and there was no recurrence on examination after 3 months.

## 4. Discussion

Peripheral nerve sheath tumours are divided into benign and malignant tumours. The benign category includes two most common and closely related tumours schwannoma or neurilemoma and neurofibroma. The other rare benign tumours include neurothekeoma, perineurioma, granular cell tumour (GCT), mucosal neuroma, and palisaded encapsulated neuroma.

Neurofibroma is defined as a well-demarcated intraneural or diffusely infiltrative extraneural tumour consisting of a mixture of cell types including Schwann cells, perineurial-like cells, and fibroblasts [2]. Neurofibromatosis (NF) was first described in 1882 by the German anatomopathologist von Recklinghausen. Two clinically and genetically distinct subtypes were identified and have been designated as NF-1 and NF-2. The most common form is NF-1 which constitutes 80–95% of the cases.

National Institute of Health consensus Development Conference in 1988 proposed a diagnostic criterion for neurofibromatosis type 1, if a patient has two or more of the following findings:

1. six or more cafe au lait macules;
2. two or more neurofibromas of any type or one plexiform neurofibroma;
3. freckling in the axillary or inguinal regions;
4. optic glioma;
5. Lisch nodules;
6. distinctive osseous lesion such as sphenoid dysplasia;
7. family history of first-degree relative with neurofibromatosis.

In the case which we reported patient showed a palatal neurofibroma, Lisch nodules, and a positive family history of first-degree relative with neurofibromatosis.

Neurofibromas in the oral cavity often involve the trigeminal and upper cervical nerves [5]; the head and neck is commonly involved because of rich innervations of this area. Involvement of superficial soft tissue is more frequent than the deeper location. In the oral cavity neurofibroma is reported to occur on tongue, lip, palate, gingiva, major salivary glands, and maxillary bones. Majority of intraosseous forms are reported in posterior mandible and few in maxilla due to the thick bundles of inferior alveolar nerve [6]. The plexiform variant of neurofibroma associated with neurofibromatosis I seems to have a significant increased incidence of malignant transformation when compared to other variants [3]. It is commonly disfiguring, causing cosmetic abnormalities, pain, functional deficits, and neurologic manifestations. On CT scan, neurofibroma appears as well-defined, oval, spherical, or fusiform masses, centered at the anatomical location of a cranial, spinal, anatomical, or peripheral nerve with displacement of adjacent muscle and blood vessels. On contrast-enhanced CT, half of neurofibromas appear homogenously hypodense. MRI exhibits a target-like appearance composed of peripheral high signal intensity with a central area of low signal [1]. On microscopy, neurofibromas have a loose myxoid background with a low cellularity. They consist of poorly organized mixture of nerve fibrils with extensive interlacing of the nerve tissue. Small axons may be seen among the proliferating Schwann cells and perineural cells. These distorted masses of myxomatous peripheral nerve are still contained within perineurium and surrounded by neurofibroma [7]. The ultrastructural findings show varying mixtures of cells with predominant Schwann cells which are surrounded by basal lamina [8]. It shows positive immunoreactivity to S-100 and CD57 (Leu-7) [1].

Lisch nodules are melanocytic hamartomas and are well-defined, avascular, smooth, regular, dome-shaped elevations, varying in colour from yellow to brown, also varying in size and number. They are predominantly located on surface of the iris. They develop during childhood and their prevalence increases with age. Histological and ultrastructural studies on Lisch nodules revealed three main cell types: pigmented cells, fibroblast-like cells, and mast cells, which resemble the neurofibroma cell population [9].

Distinguishing between isolated neurofibroma and those associated with NF-1 is important because of difference in clinical behaviour, treatment, and prognosis. The current treatment of neurofibroma is complete resection. These tumors are nonradiosensitive and limited benefit had been observed with chemotherapy. The recurrence is seen in as many as 20% of the patients with a neurofibroma after complete resection and increases to 44% with subtotal resection [10].

## 5. Conclusion

We reported a case of neurofibroma of the palate which is relatively a rare benign tumour of the oral cavity associated with neurofibromatosis type I. The diagnosis of the lesion was made based on the presence of clinical findings, positive family history, histopathology, and immunohistochemistry. Excisional biopsy of the intraoral neurofibroma was done and patient was kept under periodical observation and review and there was no recurrence after 3 months.
